# Editorial Department

**Published:** 1855-06

**Authors:** 


Dr. Oliver Wendell Holmes did not attend the late meeting of the American Medical Association. Expecting, however, that the occasion would be signalized by a public dinner, he sent on in lieu of himself the following poetical toast. But as there was no Dr. Holmes, so there was no dinner, and the neat sentiment below, instead of being ushered into the world with the cheers of hundreds of voices, and the popping of numberless champagne corks, finds quiet birth and utterance in our otherwise prosy pages:

A triple health to Friendship, Science, Art,
From heads and hands that own a common heart!
Each in its turn the other's willing slave:
Each in its season strong to heal and save.

Friendship's blind service, in the hour of need,
Wipes the pale face — and lets the victim bleed.
Science must stop to reason and explain;
ART claps his fingers on the streaming vein.

But Art's brief memory fails the hand at last;
Then SCIENCE lifts the flambeau of the past.
When both their equal impotence deplore,—
When Learning sighs, and Skill can do no more,
The tear of FRIENDSHIP pours its heavenly balm,
And soothes the pang no anodyne may calm!

